# Success of a weight loss plan for overweight dogs: The results of an international weight loss study

**DOI:** 10.1371/journal.pone.0184199

**Published:** 2017-09-08

**Authors:** John Flanagan, Thomas Bissot, Marie-Anne Hours, Bernabe Moreno, Alexandre Feugier, Alexander J. German

**Affiliations:** 1 Royal Canin Research Center, Aimargues, France; 2 Institute of Ageing and Chronic Disease, University of Liverpool, Neston, Cheshire, United Kingdom; 3 Institute of Veterinary Science, University of Liverpool, Neston, Cheshire, United Kingdom; International Nutrition Inc, UNITED STATES

## Abstract

**Introduction:**

Obesity is a global concern in dogs with an increasing prevalence, and effective weight loss solutions are required that work in different geographical regions. The main objective was to conduct an international, multi-centre, weight loss trial to determine the efficacy of a dietary weight loss intervention in obese pet dogs.

**Methods:**

A 3-month prospective observational cohort study of weight loss in 926 overweight dogs was conducted at 340 veterinary practices in 27 countries. Commercially available dry or wet weight loss diets were used, with the initial energy allocation being 250–335 kJ/kg target body weight^0.75^/day (60–80 kcal/kg target body weight^0.75^/day) depending on sex and neuter status. The primary outcome measure was percentage weight loss; the main secondary outcomes were changes in activity, quality of life, and food-seeking behaviour, which were subjectively determined from owner descriptions.

**Results:**

At baseline, median (range) age was 74 (12 to 193) months and median body condition score was 8 (range 7–9). 896 of the 926 dogs (97%) lost weight, with mean weight loss being 11.4 ±5.84%. Sexually intact dogs lost more weight than neutered dogs (*P* = 0.001), whilst female dogs lost more weight than male dogs (*P* = 0.007), with the difference being more pronounced in North and South American dogs (median [Q1, Q3]: female: 11.5% [8.5%, 14.5%]; male: 9.1% [6.3%, 12.1%], *P* = 0.053) compared with those from Europe (female: 12.3% [8.9%, 14.9%]; male: 10.9% [8.6%, 15.4%]). Finally, subjective scores for activity (*P*<0.001) and quality of life (*P*<0.001) increased sequentially, whilst scores for food-seeking behaviour decreased sequentially (*P*<0.001) during the study.

**Conclusion:**

This is the largest international multi-centre weight loss study conducted to date in obese dogs. Most dogs lost a clinically significant amount of weight, although there were notable differences between dogs of different sex, neuter status and in different geographical locations.

## Introduction

Obesity is the most common medical disease in dogs [1], and is associated with comorbidities such as orthopaedic disease and diabetes mellitus, as well as causing metabolic derangements [2,3], altered renal function [4], and respiratory dysfunction [5]. In addition to these adverse effects on health, quality of life (QOL) is poorer in obese dogs and lifespans can be shortened [6,7]. Treatment of obesity predominantly involves feeding a purpose-formulated food in restricted quantities (e.g. dietary energy intake less than maintenance requirements) to invoke controlled weight loss [8–11], as well as increasing physical activity which can provide additional benefits [12]. Successful weight loss can lessen the impact of the comorbidities associated with obesity, for example improving mobility in the face of osteoarthritis [13], improving insulin sensitivity and reversing other metabolic derangements [2,3], as well as improving QOL [6].

Many of the first weight loss studies were conducted in research colony dogs, and reported universal success with rates of weight loss progressing at 1–2% per week [14–16]. Other studies have demonstrated successful weight loss in pet dogs with naturally-occurring obesity, although weight loss is slower than in the colony studies (mean <1% per week) [10–12], and many dogs fail to reach their target weight [11,17]. Whilst the latter studies were more representative of weight loss in pet dogs than colony studies, they were limited by the fact they were conducted at academic institutions, and were arguably less representative of weight loss regimens conducted in primary care practice. Further, most studies have focused on weight loss metrics such as rate and percentage weight loss. This limitation has been addressed in other studies that have also gathered data on other outcomes such as mobility using force plate and accelerometers [13,18,19], metabolic markers [2,3], and quality of life using validated health-related QOL questionnaires [6,20]. However, overall, most canine studies tend to be small in scope, often involving a single centre and single locality. Therefore, information is lacking on how the success of weight loss varies amongst different settings and different geographic locations.

The primary aim of this study was to conduct a large, international, multi-centre trial to determine the efficacy of a short-term dietary weight loss intervention in overweight pet dogs attending primary care veterinary practices. Secondary objectives were to determine factors associated with success of the weight loss intervention, and to determine the impact of weight loss on owner perceptions of activity, food-seeking behaviour and QOL.

## Materials and methods

### Study design

This was a prospective, multi-centre, observational cohort study of overweight dogs from various geographical regions undergoing weight loss. The study has been reported according to the Strengthening and Reporting of Observational Studies in Epidemiology (STROBE) statement guidelines (S1 Checklist) [21]. The study was organised by Royal Canin, and the protocol was first approved by the Royal Canin Ethics Committee (Permit Number: 140317–22). Owners of all participating animals gave informed consent in writing. As compensation for participating, owners were not charged for the study visits, and received some or all of the weight loss diet free of charge; individual practices decided upon the appropriate compensation to offer their clients.

### Eligibility criteria, recruitment, enrolment and exclusion

Veterinary practices from 27 different countries (Table 1), from 3 global regions (Americas, Asia, and Europe), were invited to participate in the study. The scientific communication manager of the local subsidiary of the petfood supplier made initial contact and explained the nature of the trial. If the practice was interested in participating, their eligibility was checked. Practices were eligible if their veterinary staff were confident about discussing obesity and weight management with clients, and if a reliable internet connection was available (because of the requirement to use internet-based software to monitor programmes). All study investigators at the practice had to be either a qualified veterinarian or veterinary nurse (technician). The final decisions on practice participation were made between January 2015 and April 2015. After being approved for the study, participating practices recruited cases within a period of 4 months, with the weight loss programme for each dog lasting 3 months. Therefore, all cases had completed the study by the end of August 2015.

**Table 1:** Details of participating regions and countries.

Region	Country	Number of Practices	Number of dogs
Americas			
	Argentina	25	92
	Brazil	6	31
	Canada	6	16
	Chile	2	5
	Mexico	2	2
	USA	27	150
Asia			
	China (Hong Kong)	1	1
	India	3	7
	Indonesia	1	2
	Malaysia	4	8
Europe			
	Czech Republic	1	6
	Denmark	6	19
	Finland	8	17
	France	3	7
	Germany	71	145
	Greece	4	5
	Hungary	9	22
	Italy	99	178
	Latvia	2	3
	Netherlands	3	33
	Norway	5	13
	Portugal	8	73
	Romania	1	14
	Russia	2	2
	Slovakia	6	13
	Spain	15	40
	United Kingdom	11	18
Unknown ^1^	---	4	4

^1^ For 4 dogs, no practice or country information was recorded.

To make owners aware of the study, posters were displayed prominently in practice waiting rooms. Owners of overweight dogs were determined to be eligible for the study if they consented to their dog participating, they agreed to bring their dogs back for assessment, and if they were readily contactable by telephone or email. Dogs were eligible for enrolment if they were overweight (e.g. body condition score [BCS] of ≥ 7/9, see below) and were adult age, but otherwise determined to be in good health. Further, female dogs could not be pregnant or lactating at the time of enrolment. Additional eligibility criteria at the time of enrolment included the dog having no prior history of an adverse reaction to food, not requiring a therapeutic diet (other than a weight loss diet), and not having a significant concurrent disease (e.g. hypothyroidism, systemic disease such as diabetes mellitus, chronic kidney disease etc) determined by owner history, physical examination, and a review of case records. In addition, concurrent therapy with drugs that might feasibly influence the weight loss process (e.g. glucocorticoid therapy, anticonvulsants, appetite stimulants, antibacterials, insulin) was not allowed. However, prophylactic treatments such as vaccination, ecto- and endo-parasiticides were allowed. Finally, all dogs had to be used to consuming either a commercial dry or wet food exclusively or a mix of wet and dry food.

After enrolment, all reasons for subsequent exclusion were recorded. Reasons for suspending participation included failure to return for appointments, refusal and food aversion resulting in non-consumption of the diet, and failure of the owner to comply with the protocol. Development of an unrelated illness during the study was also another reason for possible trial suspension; in such cases, the attending veterinarian assessed the dog and decided whether the trial should be discontinued, with decisions being based upon the nature and severity of the illness, the treatment required, welfare of the patient and wishes of the owner. If dogs that developed an illness could continue, short-term treatments were permissible, including non-steroidal anti-inflammatory drugs and antibacterials, but all details were recorded.

### Weight loss diets

The diets used were high protein high fibre weight loss diets (Table 2), and three options were available, two of which were dry diets (dry food 1, Satiety Weight Management; dry food 2, Satiety Small Dog; Royal Canin, Aimargues, France), and the third was a wet diet (wet food, Satiety Wet, Royal Canin, Aimargues, France). All diets were designed to be complete and balanced for all essential nutrients even when fed at an energy intake that would invoke weight loss (e.g. less than maintenance requirements). However, they varied in their formulation (e.g. content of moisture, fibre, protein and nitrogen-free extract; Table 2). Dogs were fed either dry food exclusively, wet food exclusively, or a mix of wet and dry food depending upon the preferences of both owner and dog. Although either diet could be used in any dog, dry food 2 was specifically designed for feeding to small dogs (i.e. dogs <10 kg). The trial was open label in that diets were provided in their normal packaging, and no attempt was made to blind owners to their identity.

**Table 2:** Average composition of diets for weight loss.

*Criterion*	Dry food 1 ^a^		Dry food 2 ^b^		Wet food ^c^	
*ME content*	2595 kcal/kg		2670 kcal/kg		600 kcal/kg	
	Per 100g AF	g/1000 kcal (ME)	Per 100g AF	g/1000 kcal (ME)	Per 100g AF	g/1000 kcal (ME)
*Moisture*	9.5	37	9.5	36	83	138
*Crude protein*	30	116	30	112	8.5	141
*Crude fat*	9.5	37	9.5	36	2	33
*Starch*	17.5	67	16.5	62	1.8	30
*NFE*	28.8	111	28.8	108	3	50
*Crude fibre*	16.5	64	15.5	58	2	33
*TDF*	27.8	107	27.8	104	3.2	53
*Ash*	5.7	22	6.7	25	1.5	25
*Fibre sources*	Cellulose, beet pulp, FOS, psyllium husk, diet cereals		Cellulose, chicory pulp, FOS, psyllium husk, diet cereals		Cellulose, beet pulp, carrageenan, xanthan, diet cereals	
*Kibble shape*	Round (pastille)		Round (pastille)		---	

^a^ High protein high fibre dry food (Satiety Weight Management, Royal Canin);

^b^ high protein high fibre dry food (Satiety Small Dog, Royal Canin);

^c^ High protein high fibre wet food (Satiety Wet, Royal Canin);

ME = Metabolisable energy content, calculated using a predictive equation based on TDF; AF = as fed; DM = dry matter; FOS = fructo-oligo-saccharides; NFE = nitrogen-free extract; TDF = total dietary fibre.

### Measurements

All dogs were weighed by study investigators at their veterinary practice on electronic scales designed for the purpose. The make and model of the weigh scales varied depending upon the practice, but the same set of scales was always used for the same dog during its weight loss programme. Body condition was determined at inclusion using a 9-integer unit BCS system, which was a modification of the original system of Laflamme [22]. This modified system was developed with reference to an image archive of dogs attending the Royal Canin Weight Management Clinic, University of Liverpool [23], and comprising a set of 5 size-specific BCS charts, for small, medium, large, and giant breeds, respectively. The system was subsequently validated by comparing estimates of BCS with body fat mass measured by dual-energy x-ray absorptiometry as part of a separate study involving a group of thirty dogs of various breeds assessed at the Small Animal Teaching University of Liverpool, UK and a high positive correlation was observed (R_s_ = 0.74, P<0.001). Data from this study have been presented as a research communication at an international congress [24]. To ensure that study investigators were experienced in assessing body condition using the BCS system, training was provided by the local scientific communication manager to a single veterinarian professional during enrolment of the clinic into the study.

Activity and QOL were subjectively assessed by the veterinarian after a discussion with the owner (Table 3) to determine which activity and QOL descriptions were most accurate for their dog. The categories were designed to be mutually exclusive, and at least two characteristics from a category were required for it to be selected. Food-seeking behaviour was also determined subjectively by the investigator after a discussion with the owner. The different types of food-seeking behaviour (Table 3) were explained to the client and, if present, their relationship to feeding determined. Once again, to ensure consistency, the same study investigator performed scores in the same dog.

**Table 3:** Criteria for subjective determination of activity, quality of life and food seeking behaviour in study dogs.

Assessment	Scoring	Description
Activity	-1	My dog is not active
		My dog spends most of the time sleeping
		My dog is rarely playful when going out for a walk
		My dog tends to drag behind me during walks
		My dog seems to have reduced mobility e.g. is reluctant to run or to climb stairs
		My dog seems to have joint pain and stiffness
		My dog is often out of breath after short periods of exercise
	0	My dog seems normally active to me
		My dog is a bit playful
		My dog can climb to higher places but slowly
		My dog is out of breath only after long periods of exercise
	+1	My dog is very active inside and outside the household
		My dog is very playful
		My dog enjoys exercising
Quality of life	-1	My dog seems anxious and is not enjoying life
		My dog is not playful when stimulated
		My dog does not respond to orders and she/he does not learn new things
		My dog spends most of her/his time sleeping
		My dog can be irritable and/or aggressive towards some people and/or pets
		My dog has trouble climbing stairs or any high places
		My dog has mobility issues e.g. joint pain, stiffness, out of breath after exercise
		My dog seems shy e.g. avoids interaction with people and/or pets
	0	My dog does not seem anxious
		My dog occasionally shows one of the signs described in score “-1” but always limited in duration and frequency
	+1	My dog seems to enjoy life
		My dog does not show any signs included in score “-1”
Food seeking behaviour		Descriptions of food-seeking behaviours: My dog acts as if they are hungry My dog consumes food very rapidly and 'greedily' My dog vocalises (e.g. barks and/or whines) for more food My dog attempts to steal food, to steal from the dustbin, and/or to open doors of cupboards containing food My dog wakes me up at night asking to be fed My dog follows me everywhere and appears 'clingy' My dog very often turns over their bowl after meals or between meals My pet is irritable and sometime aggressive
	0	One or more food-seeking behaviours observed just before meals
	- 1	One or more food-seeking behaviours observed just before meals, and occasionally between meals
	- 2	One or more food-seeking behaviours observed just before meals, often between meals, and right after meals
	- 3	One or more food-seeking behaviours observed just before meals, constantly between meals, and right after meals

### Weight loss protocol

The study involved an initial 4-month period for case enrolment, followed by a period of approximately 3-months to enable all dogs to complete the 5-visit study protocol (Table 4). During this time, dogs attended for 5 visits, an initial assessment and enrolment visit (visit 1) and 4 follow-up visits (visits 2 to 5).

**Table 4:** Details of the 3-month study protocol.

Visit	1	2	3	4	5
Time (weeks)^1^	0	2	4	8	12
Body weight	X	X	X	X	X
Body Condition Score^2^	X				
Target Body Weight	X				
Initial Energy Allocation	X				
Allocation Adjustment		X	X	X	
Activity	X	X	X	X	X
Quality of Life	X	X	X	X	X
Food-seeking behaviour	X	X	X	X	X

^1^ Recommended time of visits in weeks as per the study protocol. Activity, quality of life and food-seeking behaviour subjectively determined based on owner descriptions (see Table 3).

^2^ Veterinary professionals continued to assess BCS during the study, and use it to help monitor progress, it was not formally recorded as an outcome measure.

During visit 1, dogs were assessed clinically using history and physical examination, and their eligibility was determined (see above). All dogs were weighed, their BCS assessed, and subjective assessments were made about their activity, QOL, and food-seeking behaviour. A 2-stage method was used to calculate the initial daily food ration for the study. First, an estimate of target body weight (TBW) was determined by dividing the current body weight by a factor that took into account the estimated percentage of excess weight (assumed to be 10% per unit of BCS between 5 and 9) [25]. Therefore, the estimated TBW for dogs with BCS 7, 8, and 9, were calculated by dividing the current body weight by 1.2, 1.3 and 1.4, respectively. The initial daily energy allocations ranged from 251 to 335 kJ per kg^0.75^ of TBW (60 to 80 kcal per kg^0.75^ of TBW) per day, depending upon sex and neuter status (sexually intact males 251 kJ/kg^0.75^ TBW [80 kcal /kg^0.75^ TBW]; neutered males 293 kJ/kg^0.75^ TBW [70 kcal/kg^0.75^ TBW]; sexually intact females 293 kJ/kg^0.75^ TBW [70 kcal/kg^0.75^ TBW]; neutered females 251 kJ/kg^0.75^ TBW 60 kcal/kg^0.75^ TBW). These allocations were adapted from energy intakes known to induce controlled weight loss in previous studies using the same or similar diets and involving obese client-owned dogs [9,10]. A previous study, presented as a research communication at an international congress, has demonstrated that this 2-stage method better predicts starting energy intake than methods that utilise current body weight (rather than TBW), that base intake on resting energy requirement, and that do not factor in sex or neuter status [26]. To ensure accuracy and consistency amongst practices and investigators, all TBW and energy allocations were calculated using an internet-based computer programme specifically developed for the trial (VET FOLLOW UP, Royal Canin). Study investigators entered details of sex, neuter status, current body weight, and BCS, and the programme then automatically calculated TBW and starting food allocation, as well as converting the starting allocation to a daily ration (in grams or tins of food), based on the food type selected (e.g. dry or mix of wet and dry food). For dry food, in all countries apart from the USA, portion size was accurately determined using electronic gram scales wherever possible. If owners refused to use electronic gram scales, a calibrated measuring cup was instead used: here, the cup was individually calibrated by first weighing a portion of food on gram scales at the practice, and marking portion size on the cup using an indelible marker pen. For the USA, owners were provided with an 8 US fl oz cup (237 mL) with marked graduations corresponding to one quarter, one third, one half, three-quarters and one cup. All food portions were provided in ounces, and a conversion provided for the corresponding number of cups. The owners were instructed to divide the daily ration into at least two daily meals, with food given in the morning and evening. All owners were counselled about not giving extra food (e.g. table scraps and treats), but formal guidelines were not provided.

The four follow-up visits were scheduled for 2 weeks (visit 2), 4 weeks (visit 3), 8 weeks (visit 4), and 12 weeks (visit 5). Veterinarians were asked to schedule each visit at these time points if possible, although some flexibility was allowed if the owner missed an appointment or if the owner could not attend at the specific times. However, for some of the statistical analyses, only data from dogs that complied most closely with the visit schedule were used (see below). At each recheck, body weight was recorded (as the primary outcome measure), and the subjective assessments of activity, QOL and food-seeking behaviour were completed. However, whilst veterinary professionals continued to assess BCS during the study, and use it to help monitor progress, it was not used as a study outcome measure given its relative insensitivity for detecting changes in body composition, (whereby a change of 10–15% body weight is typically required for a 1-unit change in BCS) [25]. The weight loss programme was also discussed with the owner to determine if the owner had been compliant with the programme, for example whether they had measured food accurately and whether the dog had obtained additional food (e.g. treats or table scraps fed by the owner or food stolen by the dog). The same internet-based computer software was also used to assist for monitoring progress of weight loss. The study investigator entered the body weight at the visit, and the software automatically calculated the rate of weight loss based upon the percentage of initial body weight lost per week. The software then suggested adjustments to the feeding plan to aim for a weekly weight loss of between 1% and 3% per week. For example, if the rate of weight loss had been <1% per week, a 10% reduction in energy allocation was suggested, whilst a 10% increase in energy allocation was recommended if weight loss had been faster than 3% per week. However, the attending veterinary professional could use their judgement on adjusting the ration, based upon the individual circumstances of the case. Examples of reasons for overriding the recommended adjustment included evidence of non-compliance (which could be corrected by owner counselling) and an owner being reluctant to change the food allocation.

### Data handling and statistical analysis

A formal sample size calculation was not performed. Instead, enrolment of practices for the trial was a pragmatic one, whereby as many dogs as possible were recruited, if they were willing to participate and met the eligibility criteria. Within each practice, a pragmatic approach was also taken for recruitment of dogs. In signing up, no targets were set for each practice but, instead, as many dogs as possible were recruited in the time limit.

The internet-based software (VET FOLLOW UP, Royal Canin) used by study investigators to determine food allocations, was also used for data recording. Data included age, sex, neuter status, breed, body weight, BCS, size, and owner-reported behaviour (activity, QOL and food-seeking). For size, dogs were assigned to one of three categories based upon TBW data (small <10 kg; medium 10–25 kg; large >25 kg). Continuous data are reported as mean ±SD, median and interquartile range (IQR), or median and range, as indicated. The restricted maximum likelihood (RML) method was applied to linear mixed models to deal with missing data. Age data were missing for 3 dogs, information on QOL was missing from 1 dog (visit 5), and information on food-seeking behaviour was missing from 1 dog (visits 1, 2, 3 and 5). Energy allocation data were not reported for a total of 80 instances in 31 dogs, whilst body weight measurements of 16 dogs were missing (7 at Visit 2, 5 at Visit 3 and 6 at Visit 4). Data were analysed with SAS v9.3 (SAS Institute Inc., Cary, NC, USA), and the level of statistical significance was set at *P*<0.05, for two-sided analyses. Datasets containing all study data are available in the supporting information (S1 and S2 Datasets).

**Fig 1:**
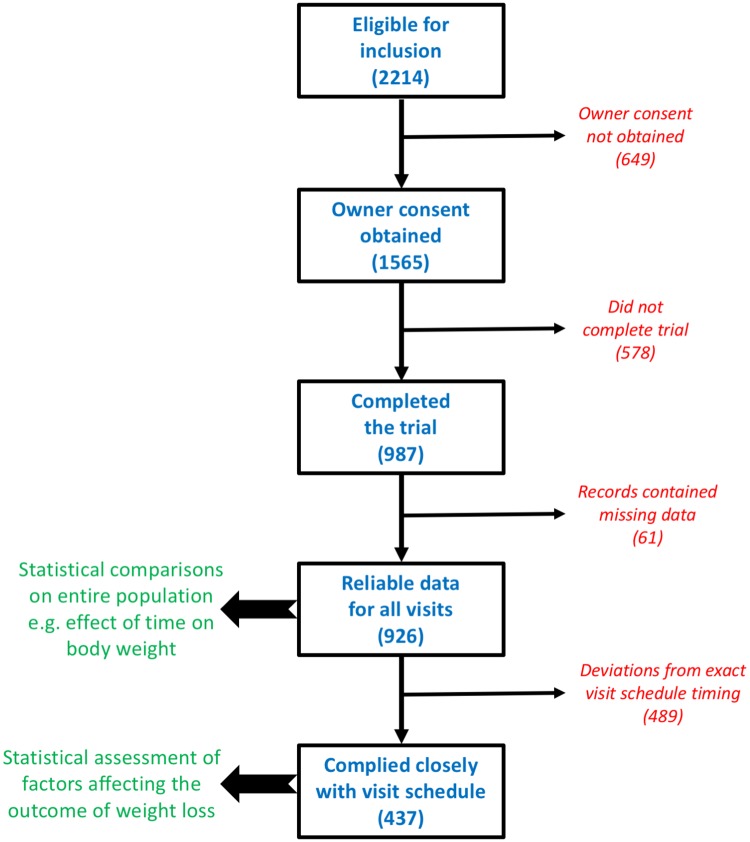
Flow diagram illustrating the number of dogs eligible and participating in the study.

Descriptive statistics on outcomes were calculated for the dogs with complete and reliable weight loss data (926 dogs; S1 Dataset). Comparative statistical analyses then performed on a subpopulation of 437 dogs that had complied best with the study visit schedule (Table 4, Fig 1, S2 Dataset). Changes in rate of weight loss were assessed using linear mixed models with visit number as a fixed effect and dog as a random term. If residuals were not normally distributed, quantitative variables were rank transformed. Given that several visits were considered, post-hoc analyses were adjusted with Scheffé’s method to avoid alpha-risk inflation.

Stepwise linear regression analyses were then performed to assess the impact of various fixed effects (age, sex, neuter status, dog size [small {<10 kg} vs. medium {≥10 to <25 kg} vs. large categories {≥25kg}], initial BCS, average energy intake during the study, and continent [Americas vs. Europe]) on percentage body weight loss in the subpopulation of 437 dogs. Contingency tables were used to assess the independence of each factor vis-à-vis all other factors. Stepwise regressions were set in such a way that each forward selection step could be followed by a backward elimination step if necessary using the Schwarz Bayesian Information Criterion (SBC) [27,28]). In this model, if removal of any effect yields a model with a lower SBC statistic than the current model, then the effect producing the smallest SBC statistic is removed. When the removal of any effect increases the SBC statistic then, provided that adding some effect lowers the SBC statistic, the effect producing the model with the lowest SBC is added (SAS v9.3; SAS Institute Inc.). The cut-off value (*P* value) to enter or remove a variable at each step was set at 0.05. Complementary analysis of baseline data was performed on the subpopulation (n = 437) using either a classic general linear model (GLM) to assess the effect of the most relevant factors identified in the stepwise regression (e.g. continent, sex and neuter status) and their interactions (e.g. sex*neuter, sex*continent, neuter*continent, and sex*neuter*continent). For the GLM analysis, residual distributions were checked for normality and were subsequently rank transformed if not normal.

Statistical analyses of subjective scores of activity, QOL and food-seeking behaviour (rank transformed to be treated as ordinal data) were conducted using linear mixed models with visit number, initial body condition (BCS 7/9 vs BCS 8-9/9) and their related interaction as fixed effects, and dog as a random term. Given that several visits were considered, post-hoc analyses were adjusted with Scheffé’s method to avoid alpha-risk inflation.

## Results

### Details of participating countries, practices, and dogs

A total of 340 veterinary practices from 27 countries participated (Table 1). After initial screening, 2214 dogs were eligible, but only 1565 of owners consented to their dog participating (Fig 1). Of the participating dogs, 987 (63.1%) completed the full 3 months of weight loss attending all visits. Of the 578 dogs that did not complete the trial, 474 dogs (82.0%) failed to return for revisits and were lost to follow up, 40 (6.9%) stopped due to owner non-compliance, 11 (1.9%) stopped because of a concurrent medical condition, 7 (1.2%) stopped because the dog refused to eat the food, and 2 (0.2%) owners found the programme too tough to follow. The reasons why the remaining 44 dogs (7.6%) stopped were not recorded.

After reviewing the records, complete and reliable data for all 5 visits were available for 926 of the 987 dogs, 568 (61%) of which were female (433 neutered) and 358 dogs (39%) were male (238 neutered). The dogs comprised 82 different breeds, with 297 dogs (32%) being of mixed or unknown breed (Table 5). The most common pedigree breeds represented were Labrador Retriever (142, 15%), Golden Retriever (60, 6%), Beagle (37, 4%) Pug (34, 4%), Dachshund (30, 3%), Chihuahua (24, 3%) and German Shepherd dog (24, 3%). Age data were available for 903 dogs and, within this group, the median age was 74 months (range 12 to 193 months). Median starting body weight of the dogs was 23.2 kg (range 2.1 to 80.0 kg) and median BCS was 8 (range 7 to 9).

**Table 5:** Details of the study dogs with complete and reliable weight loss data.

Variable	All dogs ^1^	Most compliant dogs ^2^
**Number**	926	437
**Sex**		
Entire male	120 (13%)	47 (11%)
Neutered male	238 (26%)	128 (29%)
Entire female	135 (14%)	58 (13%)
Neutered female	433 (47%)	204 (47%)
**Age (months)**	74 (12 to 193)	72 (12 to 166)
**Breed**		
Mixed breed	297 (32%)	132 (30%)
Labrador retriever	142 (15%)	47 (11%)
Golden retriever	60 (6%)	33 (8%)
Beagle	37 (4%)	21 (5%)
Pug	34 (4%)	12 (3%)
Dachshund	30 (3%)	17 (4%)
Chihuahua	24 (3%)	15 (3%)
German shepherd dog	24 (3%)	5 (1%)
Other breeds	278 (30%)	155 (35%)
**Weight (kg)**	23.2 (2.1 to 80.0)	22.0 (3.0 to 80)
**Body condition score** ^3^	8 (7 to 9)	8 (7 to 9)

^1^ The 926 dogs that completed the 3-month weight loss trial;

^2^ The 437 dogs that complied best with the visit schedule;

^3^ Body condition score determined using a 9-integer unit system [22].

### Weight loss food and dietary energy allocation

Of the 926 study dogs, 750 (81%) were fed dry food exclusively (median weight 25.5 kg, range 2.1–80.0 kg), 170 (18%) were fed a mix of wet and dry food (median weight 16.5 kg, range 2.6–72.9 kg), and the remaining 6 (1%) were fed wet food exclusively (median weight 40.7 kg, range 3.5–54.2 kg). Of the 750 dogs that were fed dry food exclusively, 601 (65% of total) were given dry diet 1 (median weight 31.8 kg, range 3.5–80.0 kg) and the remaining 149 (16% of total) were given dry diet 2 (median weight 9.0 kg, range 2.1–25.4 kg). Of the 170 dogs that were fed a mix of wet and dry food, dry food 1 and dry food 2 were used in 110 dogs (12% of total, median weight 10.2 kg, range 2.6–41.0 kg) and 60 dogs (6% of total, median weight 30.3 kg, range 4.8–72.9 kg), respectively.

The mean ±SD starting allocation for the study dogs was 276 ±35.6 kJ/kg^0.75^/day TBW (66 ±8.5 kcal/kg^0.75^/day TBW), whilst the mean energy allocation for the whole of the 12 weeks was 264 ±43.5 kJ/kg^0.75^/day TBW (63 ±10.2 kcal/kg^0.75^/day TBW).

### Weight loss outcomes

Weight loss was seen in 896/926 dogs (96.8%), whilst 14 (1.5%) maintained a stable weight (i.e. weight remained ±1% of starting body weight), and 16 (1.7%) gained >1% weight. A total of 896 (96.8%), 814 (87.9%), 543 (58.6%), and 78 (8.4%) dogs lost more than 1%, 5%, 10% and 20% of their starting body weight, respectively. By the end of the study 65/926 dogs (7.0%) had reached target body weight and were in ideal body condition, as judged by BCS performed by the attending veterinary professional.

Although the planned schedule for follow up was 4 visits over 12 weeks (visits 2 to 5), there was considerable deviation from this, with median (IQR) time from starting the programme (visit 1) being 15 days (14–19 days), 32 days (28–41 days), 61 days (56–70 days), and 91 days (82–102 days) for visits 2, 3, 4 and 5, respectively. The mean (±SD) initial body weight lost was 3.2 ±3.20% (by visit 2), 5.7 ±3.96% (by visit 3), 8.7 ±4.85% (by visit 4) and 11.4 ±5.85% (by visit 5). The mean ±SD rate of weight loss for the whole of the study was 0.9 ±0.45% of initial body weight per week, whilst rates of weight loss for the periods between visits were as follows: visit 1 to 2: 1.3% per week (0.6–1.9% per week); visit 2 to 3: 1.0% per week (IQR: 0.4–1.5% per week); visit 3 to 4: 0.9% per week (IQR: 0.4–1.2% per week); and visit 4 to 5: 0.8% per week (IQR: 0.3–1.1% per week). The number of dogs losing weight at >2% per week was 221 (23.9%) between visits 1 and 2, 105 (11.3%) between visits 2–3, 32 (3.4%) between visits 3–4, and 9 (1.0%) between visits 4–5. Only 6 dogs (0.6%) maintained a rate of weight loss of >2% per week throughout the whole study. In the 437 dogs (47.2%) that complied closely with the visit schedule, mean (±SD) rate of weight loss decreased sequentially over the 5 visits (visit 1 to 2: 1.4±1.35% per week; visit 2 to 3: 1.1 ±1.26% per week; visit 3 to 4: 0.9 ±0.84% per week; visit 4 to 5: 0.7 ±0.96% per week; *P*<0.001).

### Factors affecting outcome of weight loss

**Fig 2:**
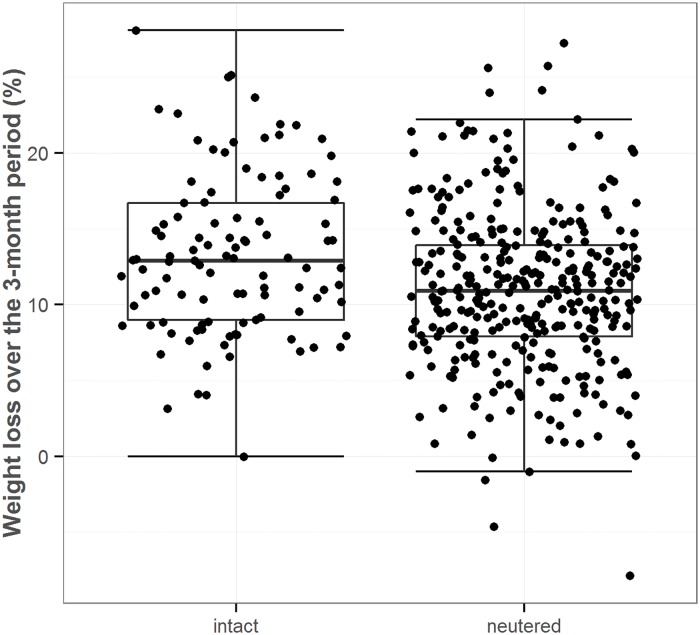
Box-and-whisker dot plots comparing percentage weight loss in intact and neutered dogs over the 3-month study. The boxes depict median (horizontal line) and inter-quartile range (top and bottom of box), the vertical lines depict 1.5 times the inter-quartile range, and individual dogs are shown by the black circles.

**Fig 3:**
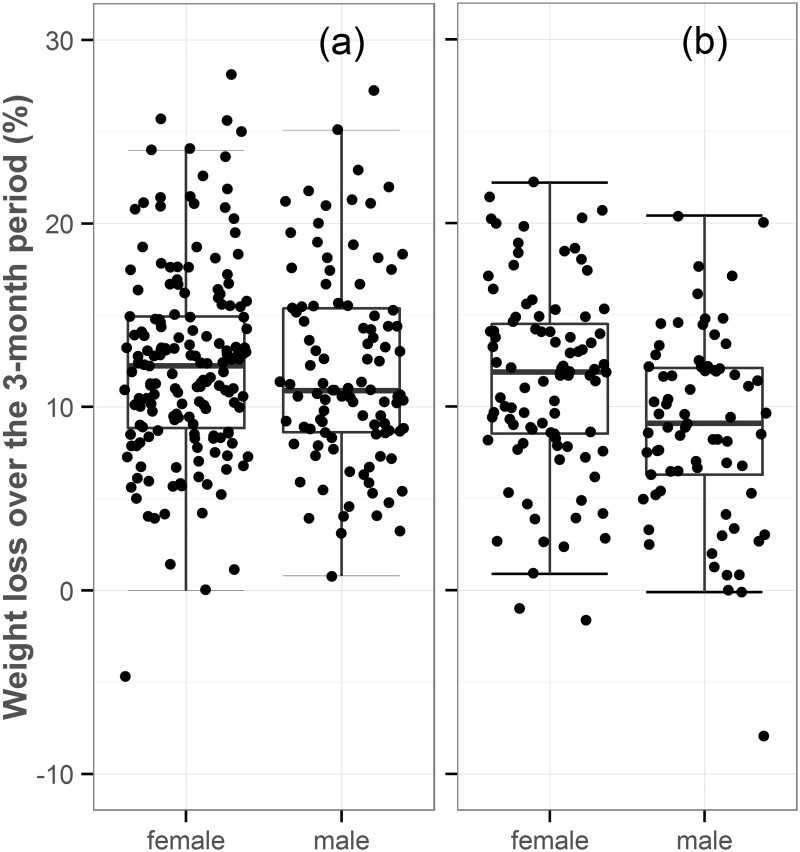
Box-and-whisker dot plots comparing percentage weight loss over the 3-month study in male and female dogs in Europe (a) and the Americas (b). The boxes depict median (horizontal line) and inter-quartile range (top and bottom of box), the vertical lines depict 1.5 times the inter-quartile range, and individual dogs are shown by the black circles.

The 437 dogs (47.2%) that complied closely with the visit schedule were also used to determine factors influencing the percentage weight loss, the primary outcome of the study. Inclusion of the terms sex, neuter status and continent decreased the SBC and so were retained for analysis using GLM; in contrast, the presence of the remaining factors (age, size, initial BCS and average calorie allocation) had a negative effect on the overall fit of the final model, and so were not retained. Using GLM, sex (*P* = 0.007) and neuter status (*P* = 0.001) were independently associated with percentage weight loss, with female dogs (median 12.0% weight loss, IQR 8.6–14.9%) losing more weight than male dogs (median 10.4% weight loss, IQR 7.4–14.1%), and entire dogs (median 12.9% weight loss, IQR 9.0–16.7%) losing more weight than neutered dogs (median 10.9% weight loss, IQR 7.9–14.0%, Fig 2). Post-hoc analyses showed that at baseline, neuter status was homogenously distributed for BCS, sexual status and age levels, whereas it was heterogeneously distributed for continent and size levels. Although continent was not significantly associated with percentage weight loss in this model (*P* = 0.216), the interaction of sex and continent was (*P* = 0.030, Fig 3). In this respect, the sex effect on weight loss was most pronounced on the American continent (female median 11.5%, IQR 8.5–14.5%; male median 9.1%, IQR 6.3–12.1%, p = 0.053), compared to the European continent (female median 12.3%, IQR 8.9–14.9%; male median 10.9%, IQR 8.6–15.4%, *P* = 0.957). Post-hoc analyses of sexual status at baseline revealed that it was homogenously distributed for all other considered factors. No other interactions (neuter*sex, *P* = 0.508; neuter*continent, *P* = 0.154; neuter*sex*continent, *P* = 0.205) were significant in the final model.

### Activity

**Fig 4:**
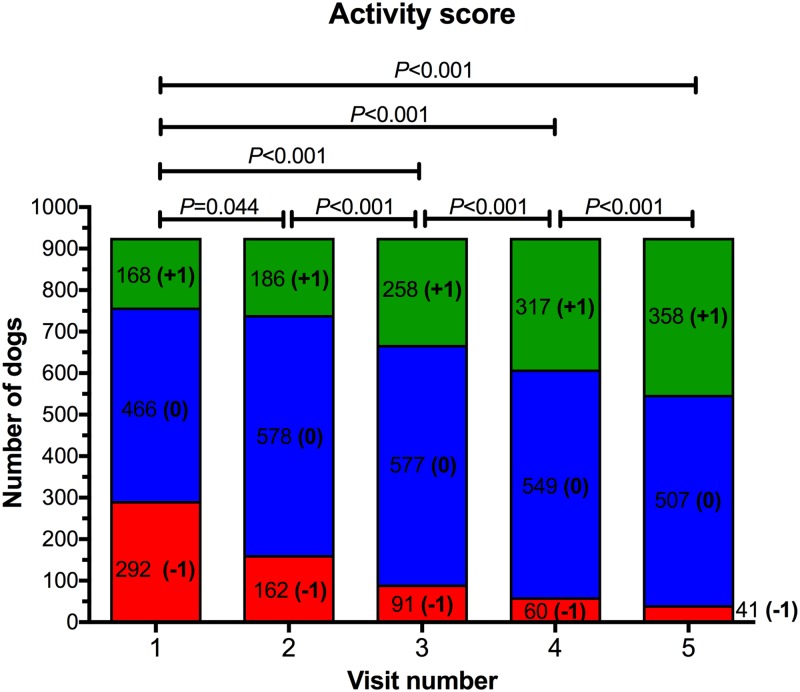
Activity scores for dogs during the study. Activity score was subjectively determined at each visit after a discussion between the veterinarian and owner (Table 3). At each visit, blocks with different colours represent the proportion of dogs assigned an activity score of -1 (red), 0 (blue) and 1 (green), respectively. The number of dogs assigned to each category is also shown.

At the initial visit, 292 (31.5%), 466 (50.3%), and 168 (18.1%) dogs had an owner-reported activity score of -1, 0 and 1, respectively (Fig 4). There was no difference in activity scores between dogs with BCS 7/9 and those with BCS 8-9/9, (*P* = 0.226). Activity increased during the study (*P*<0.001), with post-hoc analysis revealing significantly improved activity for visits 2, 3, 4, and 5, compared with baseline (Fig 4), and significant improvement in activity at each sequential visit.

### Quality of life

**Fig 5:**
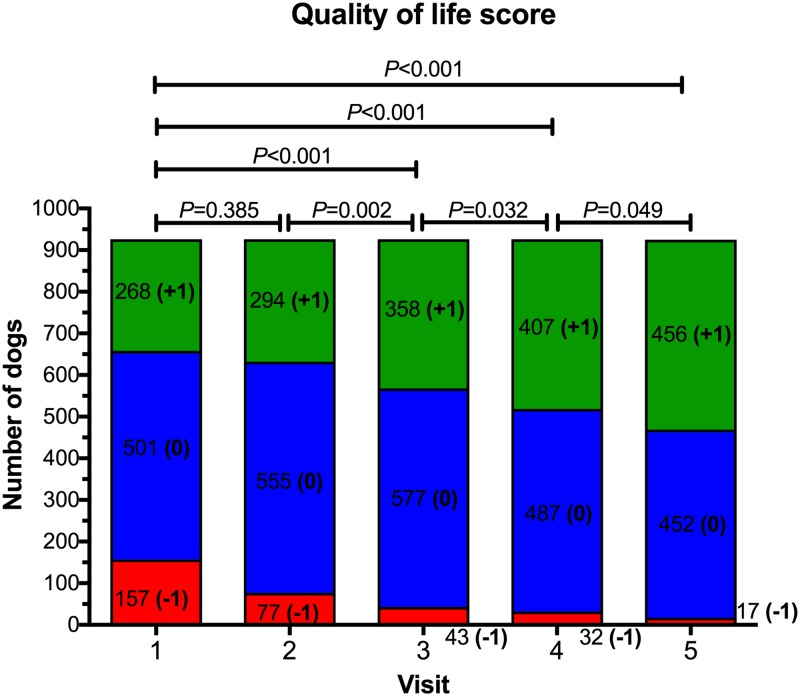
Quality of life (QOL) scores for dogs during the study. QOL was subjectively determined at each visit after a discussion between the veterinarian and owner (Table 3). At each visit, blocks with different colours represent the proportion of dogs assigned a QOL score of -1 (red), 0 (blue) and 1 (green), respectively. The number of dogs assigned to each category is also shown.

At the initial visit, 157 (17.0%), 501 (54.1%), and 268 (28.9%) dogs had an owner-reported QOL score of -1, 0 and 1, respectively, (Fig 5). There was no difference in QOL scores between dogs with BCS 7/9 and those with BCS 8-9/9 (*P* = 0.195). Quality of life increased during the study (*P*<0.001), with post-hoc analysis revealing significantly improved quality of life for visits 3, 4, and 5, compared with baseline (Fig 5). Further, apart from between visits 1 and 2, QOL improved significantly at each sequential visit.

### Food-seeking behaviour

**Fig 6:**
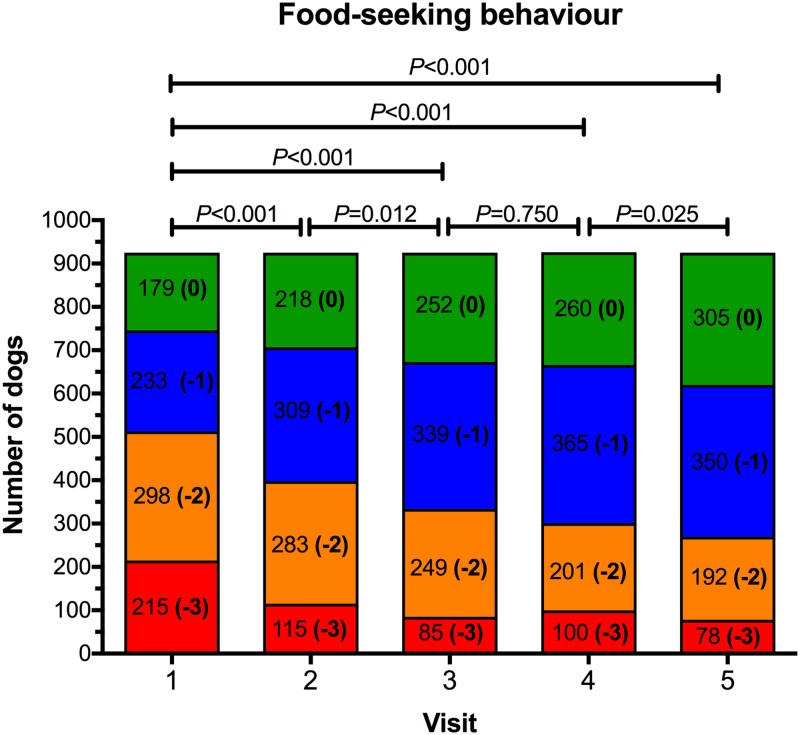
Food-seeking behaviour scores for dogs during the study. Food seeking behaviour was subjectively determined at each visit after a discussion between the veterinarian and owner (Table 3). At each visit, blocks with different colours represent the proportion of dogs assigned a food-seeking behaviour score of -3 (red), -2 (orange), -1 (blue) and 0 (green), respectively. The number of dogs assigned to each category is also shown.

At the initial visit, 215 (23.2%), 298 (32.2%), 233 (25.2%), and 179 (19.4%) dogs had an owner-reported food-seeking behaviour score of -3, -2, -1, and 0, respectively (Fig 6). There was no difference in the proportion of dogs in the different food-seeking categories between dogs with BCS 7/9 and those with BCS 8-9/9 (*P* = 0.367), but a significant association was identified for food-seeking behaviour between visit number and neuter status, whereby food intake for intact dogs was greater than neutered dogs at visit 5 only (*P* = 0.008). Decreased food-seeking behaviour was observed during the study (*P*<0.001), with post-hoc analysis revealed that food-seeking behaviour improved for visits 2, 3, 4, and 5, compared with baseline (Fig 6). Further, apart from between visits 3 and 4, food-seeking behaviour improved significantly at each sequential visit.

## Discussion

We report the largest multi-centre weight loss study conducted to date on overweight pet dogs. Most dogs enrolled completed all 12 weeks of the trial, and lost an average of 11% of their body weight. A weight loss rate of 1–2% per week is a frequently suggested target for weight loss, based upon experimental studies involving colonies of research dogs [14,15,16]. There was wide variation in the rates of weight loss amongst dogs in this study, which could be due to a variety of factors, including the method used to estimate starting energy intake. In this respect, such methods use mathematical equations to estimate the starting energy intake for weight loss; whilst such estimates correlate strongly on average with the actual energy intakes that lead to successful weight loss, they can either over- or under-estimate the requirements of some dogs [26]. Further, the average rate of weight loss was <1% per week, and this occurred even though adjustments to energy intake were made when weight loss was slower than expected. Although the computer software recommended a 10% reduction in intake, veterinary professionals could tailor the intake depending on the circumstances of the case. It is possible that faster rates of weight loss might have been observed had veterinary professionals been allowed to make larger adjustments in energy intake. That said, the rate of weight loss observed in the current study is similar to the rates of weight loss seen in many other studies assessing weight loss in obese pet dogs where, again, energy intake adjustments were made according to progress [9,10,11,12]. Further, although a rate of weight loss >1% per week was not achieved by many study dogs, others fared better with approximately a quarter losing >2% per week during the early stages. In theory, such rates of weight loss could be concerning, not least given that some studies have suggested that faster rates of weight loss might increase loss of lean tissue mass in cats [29]. However, rate of weight loss has not been associated with lean tissue loss in other work [9], and most dogs did not maintain this rate of loss throughout the study. Further work would be needed to determine if such a short-term period of rapid weight loss increases lean tissue loss.

Criticisms of the weight loss studies previously conducted in obese pet dogs include the fact that they are conducted at single centres, which are often academic institutions. Whilst such studies have the advantage of uniformity in recruitment, management, weight loss regimen and follow-up, single-centre studies are usually small and therefore do not reflect the variability of weight loss within typical primary care practices. A strength of the current study is the fact that it involved many primary care practices from diverse locations and, whilst there were some differences in outcome, results were overwhelmingly positive. This suggests that the principles of weight management are appropriate for a range of clinical practices across many countries.

Three factors were identified that affected the amount of weight lost overall, sex, neuter status, and continent. To our knowledge, this is the first time that any such factors have been shown to affect outcomes of weight loss. The fact that sexually intact dogs lost more weight than neutered dogs is not unexpected, since energy requirements for neutered dogs are typically less [30,31]. That said, we had attempted to take this into account when calculating initial energy allocation, with neutered dogs being given 10 kcal per kg^0.75^ less that their sexually intact counterparts. One explanation for the slower rate of weight loss despite greater energy restriction might be because neutered dogs displayed greater food seeking behaviour, and this might have led to worse owner compliance on the programme. The reason why percentage weight loss was greater in female compared with male dogs is not clear. This might have been because the starting energy allocation for female dogs was less than for male dogs. Most intriguing is the effect of geographical location on this sex effect, whereby the difference between male and female dogs was more marked in the Americas compared with Europe. This occurred despite the same protocol for weight loss being used (e.g. food, computer software, monitoring protocol, and rules governing adjustments in food portions). Further work is required firstly to confirm this study observation, and secondly to determine its cause.

In addition to assessing short-term weight loss outcomes, we assessed owner opinions on their dog's behaviour during the weight loss trial. Quality of life was judged subjectively using an owner questionnaire. The main reason for using this method was that it was simple, enabling information to be gathered from an owner in a matter of minutes. However, its main disadvantage was the fact that it had not been previously validated. A previously-validated questionnaire could instead have been used, for example, the health-related QOL questionnaire that some of the authors have previously used [20]. However, being 19-pages long and including over 100 questions, it takes considerable time for an owner to complete, and then requires complex statistical analysis. Not only could this have created issues of compliance with owners, but the data analysis would have been problematic given the size of the current study. Despite the limitation of using an unvalidated questionnaire, findings were consistent with those of other studies assessing health-related QOL with validated methods [6, 20]. In one of these studies, 3 of 4 aspects of QOL improved significantly with weight loss, including an improvement in 'vitality' (mobility-related behaviour) and a decrease in both 'emotional disturbances' and 'pain-related behaviour' [6]. In a similar manner, the owners in the current study reported that their dogs' QOL improved after weight loss, and these improvements were observed within the first 2 weeks with further sequential improvements thereafter.

Previous work has revealed mixed results in terms of the effects of weight loss on activity. Studies using accelerometry have demonstrated decreased activity in obese compared with lean dogs [18], but have not demonstrated improvements in activity in obese dogs that lose weight [12, 19]. In contrast, other objective assessments of mobility such as force plate analysis have demonstrated both differences in gait between lean and obese dogs [32], and improvements in mobility of obese dogs with concurrent osteoarthritis after modest (i.e. just 6%) weight loss [13]. Further, including physical activity in a weight loss regimen helps preserve lean tissue mass [12]. Although the method of assessment of activity in the current study was a subjective one, the improvement in activity observed by owners is equivalent to subjective owner improvement reported in other studies where objective assessments have also been used [10,11].

Maintaining owner compliance with weight programmes is a key challenge for veterinary professionals. This is highlighted by the fact that 37% of enrolled dogs did not complete the trial, mostly for failure either to comply with the individualised programme or for failure to return for visits. Furthermore, although only 4 follow-up visits were required, there was marked variability in the timings of these visits. Whilst this may have influenced the outcomes of the study, this variability is expected with weight loss programmes in pet dogs. In many respects, this is a strength of the weight loss protocol, the fact that most dogs had a positive outcome in terms of weight loss and behavioural improvements despite such deviations in study protocol. A further challenge for owners complying with a weight loss programme is the fact that energy restriction has the potential to cause hunger and this can then lead to an increase in food-seeking behaviour [9]. Unexpectedly, most owners did not report increased food-seeking behaviour in the current study and, in fact, a significant decrease in such behaviour was noted. This observation might be because a high protein high fibre diet was used since such diets are known to reduce voluntary food intake compared with other diets [33,34], as well as improving outcomes of weight loss [10]. However, a genuine effect of diet cannot be confirmed because no comparator diet was used in the study by way of control. Instead, owners' opinions about food-seeking behaviour could have been influenced by a 'placebo effect' since the assessment of food-seeking behaviour was subjective. Owner opinions of food-seeking behaviour might have been biased by what veterinary professionals told them about the characteristics of the weight loss diet. Further, the trial was short-term and it is possible that owners would have observed more food-seeking behaviour as time went on, not least given that further reductions to energy intake would have been expected to maintain weight loss. This increasing energy restriction might increase the tendency for food-seeking behaviour over the longer term. Ideally, further studies should be considered to determine how food-seeking behaviour changes with weight loss.

One limitation of the current study was the fact that it was short term and the dogs were not followed until they achieved an ideal body condition, with few losing >15% of their starting body weight. This is not surprising since the median duration of a complete weight loss cycle (e.g. returning dogs to ideal body weight) is 9 months, and some dogs require >12 months to reach target [9]. Therefore, the dogs in the current study might have benefited more if the study had continued for longer. That said, measurable benefits have been seen in other studies where dogs lost an equivalent amount of weight; for example, improved mobility, as determined by force plate analysis, has been observed with just 6% weight loss [13]. The rate of weight loss decreased significantly during the study, which is consistent with findings observed in other studies [35]. This makes weight loss challenging for the most obese dogs with the most weight to lose, perhaps accounting for why they are more likely to fail [17]. However, whilst the short-term nature of the study is a weakness, it nonetheless highlights the fact the benefits of weight loss occur before target weight is reached. In this respect, owners reported improvements in both activity and QOL during weight loss even though few reached their target weight. That said, given the potential for failure with a complete weight loss programme, some have argued that partial weight loss programmes might be more appropriate for some dogs, and returning a dog to ideal weight might not be necessary [35]. Such strategies have the benefit that they are shorter and more realistic for many owners. Further, since they tap into the success of the early weight loss period, compliance is higher [35]. The results of the current study provide some support for this approach, since owners reported significant improvements in wellbeing even though the weight loss was only partial.

A second limitation was the fact that the study was uncontrolled and open label, in that both the veterinary professionals and owners were aware of the identity of the diets used for the study and might have been influenced, for example, by the packaging. Thus, it neither proves that the weight loss diet used was superior to other diets, nor that the findings can necessarily be assumed to apply to other weight loss diets. As alluded to above, the possibility of a placebo effect cannot be discounted either. This is unlikely to be the case for changes that were objectively measured, such as body weight, but cannot be discounted for more subjective observations, such as changes in activity, QOL, and food-seeking behaviour. In this respect, a recent meta-analysis of placebo interventions in human studies demonstrated that significant clinical effects of placebo interventions can occur for patient-reported outcomes such as pain and nausea [36], which are also subjective. Therefore, these results should be interpreted cautiously and, ideally, randomised controlled trials should be considered.

The global nature of this study was a key aspect and enabled differences in geographical location to be assessed. However, a third study limitation was the fact that no attempt was made to recruit cases in a systematic fashion to ensure that all regions were appropriately represented. This limited the study’s ability to compare factors associated with weight loss amongst all geographical regions. Instead, comparisons were only made between dogs recruited from the European and American continents. Fourth, there were differences in how food portions were measured, with some owners using gram scales, others using a calibrated measuring cup, and those from the USA using a non-calibrated measuring cup. Whilst this might have influenced outcomes, the method of measuring portions was unfortunately not recorded. A further study limitation was the fact that no information was gathered from owners about giving extra food such as table scraps and treats. Therefore, we were not able to explore whether such owner practices varied within the study population and, most notably, amongst different geographical locations. A final study limitation is the fact that many dogs were enrolled, but a significant proportion (37%) did not complete the trial. Whilst this retention rate is consistent with other field weight loss studies [11,17], it emphasises the challenges that can be faced on a weight loss programme, most notably in terms of maintaining compliance for the owner. This highlights the need for veterinary professionals to pay close attention to maintaining good communication with owners during the whole of a weight loss period, to keep them motivated and address any problems they face.

## Conclusion

In summary, this is the largest international multi-centre weight loss study conducted to date in overweight dogs. Most dogs lost weight, but there were notable differences between intact and neutered dogs, between dogs of different sexes and between dogs in different geographical locations. While the short-term duration of the study did not permit most dogs to reach their target weight, owners observed positive behavioural changes including improved activity and quality of life.

## Supporting information

S1 Checklist Checklist for the STROBE statement. The table lists the items of the respective checklist, and the location within the manuscript where they can be found.
(DOC)

S1 Dataset Complete study dataset. Computer spreadsheet containing study data for the 926 dogs in the study.
(XLSX)

S2 Dataset Dataset from the subpopulation of dogs undergoing further statistical analysis. Computer spreadsheet containing study data for the subset of 437 dogs that had complied best with the study visit schedule. These data were used to assess the impact of various factors on weight loss.
(XLSX)
